# Calculation of Target-Specific Point Distribution for 2D Mobile Laser Scanners

**DOI:** 10.3390/s140609471

**Published:** 2014-05-27

**Authors:** Conor Cahalane, Conor P. McElhinney, Paul Lewis, Tim McCarthy

**Affiliations:** National Centre for Geocomputation, Iontas, NUI Maynooth, Maynooth, Co. Kildare, Ireland; E-Mails: conormce@cs.nuim.ie (C.P.M.); paul.lewis@nuim.ie (P.L.); tim.mccarthy@nuim.ie (T.M.)

**Keywords:** mobile mapping systems, performance, LiDAR

## Abstract

The current generation of Mobile Mapping Systems (MMSs) capture high density spatial data in a short time-frame. The quantity of data is difficult to predict as there is no concrete understanding of the point density that different scanner configurations and hardware settings will exhibit for objects at specific distances. Obtaining the required point density impacts survey time, processing time, data storage and is also the underlying limit of automated algorithms. This paper details a novel method for calculating point and profile information for terrestrial MMSs which are required for any point density calculation. Through application of algorithms utilising 3D surface normals and 2D geometric formulae, the theoretically optimal profile spacing and point spacing are calculated on targets. Both of these elements are a major factor in calculating point density on arbitrary objects, such as road signs, poles or buildings-all important features in asset management surveys.

## 1. Introduction

**Figure 1.**
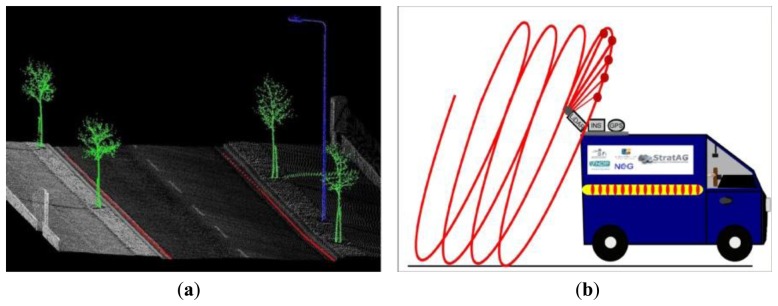
Data processing and point distribution (**a**) automated algorithms identify features (**b**) vehicle velocity and MMS hardware configuration results in a corkscrew scanning pattern.

MMSs operating laser scanners are capable of producing high density point clouds, but this results in high data volumes and increased processing times. Manually interrogating this data is extremely time consuming and therefore automated algorithms play an important role in processing. These algorithms are designed to automatically recognise features in point clouds, thus eliminating or reducing the need for manual input. Figure 1a displays an example of three automated algorithms developed by [1], which have identified trees, poles and the road edge and other examples can be seen in work by [2–7]. The distribution of points on an object influences the success of the algorithm, for example [8–10] require a minimum number of points per scan line to recognise a cylindrical object and the work presented by [11] demonstrated the importance of a high point density on spatial accuracy.

Defining point distribution is a complicated process. Hardware suppliers generally define scanner point distribution as the number of points per m^2^, yet for curved surfaces this confines a 3D value to a 2D measurement and ignores variations in point distribution across large targets. To include this third dimension, [12] projected all points per m^3^ onto a 2D circle. In [13] the authors also discussed point distribution and ways to define it for 3D data captured from multiple platforms, further reinforcing the link between point distribution and automated data processing. Part of the difficulty in defining point distribution is due to the fact that additional research is required to calculate what point distribution different platforms are capable of, particularly for project managers designing survey specifications. For example, one MMS survey specification [14] states that, 'the point density should be sufficient to identify and extract physical detail to the accuracy specified for the project' but does not specify what point density will permit that accuracy, or what MMS configuration will facilitate that point density. In another specification, [15] inform that clients requesting LiDAR surveys must carefully specify ‘the point-cloud point density’ but link this to the speed of vehicle only. In [16] this was improved significantly by subdividing point density into ‘coarse’, ‘intermediate’, and ‘fine’ and it was also recommended that a point density map with summary statistics be requested as a deliverable. It is our opinion that the distribution of the points on the surface is a possible compliment to the traditional point density definition. In [16] point distribution is explored, and the authors provide tabular information on what point spacing is required to achieve a specific point density, but do not define what MMS hardware, hardware configuration or operating parameters are required to achieve that point spacing.

**Figure 2.**
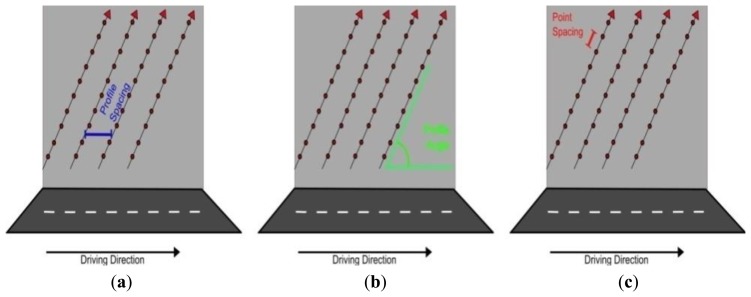
A corkscrew scan pattern intersecting with a vertical surface results in three distinctive features (**a**) profile spacing (**b**) profile angle and (**c**) the point spacing.

At present there are no generic, robust methods for quantifying point distribution and we believe the procedure set down in this paper will be the first example of this. Modern MMSs operate a 2D, full-circle laser scanner designed for mobile surveys. 2D scanners utilise the forward motion of the vehicle to provide 3D data (Figure 1b). When this scanning pattern intersects with a planar surface, the laser points are distributed over the surface in a linear pattern. These lines are termed, ‘scan profiles’. The gaps formed between each scan profile is the ‘profile spacing’ (Figure 2a). The angle of the scan profile on the target is influenced by the orientation of both the scanner and the target. This angle is termed the ‘profile angle’ and is illustrated in Figure 2b. The vehicle speed, the orientation of the scanner(s), the scanner mirror frequency (M_f_) and the orientation of the object all influence scan profiles. The distance between subsequent points along a scan profile is known as the ‘point spacing’, as illustrated in Figure 2c. Point spacing is influenced by a number of factors, including: the pulse repetition rate (PRR), which is the number of pulses per second, M_f_, which controls the number of mirror rotations per second, the range to the target from the scanner, the scanner's field of view (FOV), the height difference between target and scanner, the orientation of the target and finally the orientation of the scanner. The orientation of the scanner is important for maximising coverage of the environment and can be varied between surveys. Scan hardware settings are also variable and dependant on the smallest target in the survey specification. Accurately calculating the influence that any variation in configuration or settings has on point distribution is important for optimising MMS performance. This paper presents a novel and accurate method to do this.

Point distribution is not only of interest to MMSs. In [17] the resolution of terrestrial laser scanner (TLS) point clouds was investigated and [18,19] have investigated the smallest feature that can be recognised in a TLS point cloud. Research like this is of benefit to TLS operators surveying fine-detail like statues or paintings, but the majority of MMSs are unsuitable for these surveys. Although it is possible to mount a TLS on a vehicle and use it in ‘stop and go’ mode, these are not suitable for mobile surveys. In ‘stop and go’ a tripod mounted scanner is placed on a moving platform and driven to a survey point [20]. The vehicle stops at this point and then scans the entire scene. The vehicle then moves to the next survey point and this process is repeated. To operate a TLS on a moving platform, the TLS must have one of its axes of rotation locked so it can operate in ‘profile mode’ [21,22]. However, due to their popularity with MMS designers, e.g., the Optech Lynx [23], Trimble MX8 [24] and StreetMapper [25], the point and profile algorithms described in this paper focus on 2D full circle scanners only. Existing work in this area calculates point distribution through three main methods: manual measurements, geometric formulae and LiDAR simulations.

### 1.1. Manual Measurements

By locking one rotation axis of a FARO TLS, [22] operated in profile mode and then manually measured the profile and point spacing at different ranges, speeds and scan frequencies post-mission. It was then possible to approximate what point distribution a user could expect from that MMS at a similar survey site. In [20] the authors operated a Leica HDS in profile mode and provided a table listing profile spacing at three vehicle speeds and three M_f_. Only [22] incorporated scanner rotations. Unlike TLSs such as the Leica HDS 4500, vertical scanner rotations can be implemented with the FARO. Neither study incorporated dual-axis scanner rotations (a horizontal and a vertical rotation) or provided a method to calculate point distribution.

### 1.2. Geometric Formulae

Most laser scanner manufacturers provide information on the point density that a user can expect from their hardware. Riegl [26] provide detailed graphs plotting the point density a user can expect for various scanner, target and vehicle parameters. This is useful in the mission planning stage, however, it does not incorporate scanner rotations, target rotations or any height difference between scanner and target. These graphs are also hardware specific. These graphs are limited to specifying point density by calculating point spacing at a single target location, whereas point spacing varies over a target, something that is particularly relevant for angled surfaces and in [27] we discussed the significance of this. RiACQUIRE [28] is a useful mission planning tool that can calculate profile spacing, point pacing and point density for Riegl scanners on a target, however, RiACQUIRE does not incorporate horizontal or vertical scanner rotations, horizontal or vertical target rotations, height difference between scanner and target or different target types. Additional geometric formulae have been employed by [29] to identify the effect that vehicle direction and velocity has on scan profiles. Their work focused on automatic detection of objects and the purpose of this calculation was to eliminate areas of low point density to decrease processing time. In [30] useful formulae and graphical information are provided for calculating point spacing and point density on the road surface but limited the inputs to their formulae to the PRR, M_f_, measurement range and vehicle velocity.

### 1.3. Simulations

A LiDAR simulation models the real world interaction between a LiDAR system and the terrain. They have been used for investigating aerial platforms [31], but the viewing geometry is less complicated for aerial platforms and the FOV is more restricted. The authors of [32] designed and tested a simulator for assessing errors, algorithm development and system validation for aerial and terrestrial systems. Simulators are useful tools for assessing point clouds or for algorithm development, but do not provide a method for calculating point distribution. Although [12] included important elements in their simulator: dual-axis scanner rotations, variations in scanner location, different PRRs and different M_f_, their simulator did not calculate point distribution. Once any simulation is complete, point and profile measurements are manual and localised to a single point on a target or an average measurement is applied to the area in question and a colour scheme is applied to aid with visualisation.

## 2. Calculating Point and Profile Information

In [27] we introduced the overall system, the **M**ob**I**le **M**apping po**I**nt density **C**alculator, or MIMIC. MIMIC calculates the theoretically optimal position that each laser pulse strikes a surface at using geometric formulae. Random events such as multiple returns, occlusions or changes in the width of the laser footprint due to an increase in scan range or the orientation of the surface are not modelled in MIMIC. Although these factors may change the position or number of individual laser pulses, the purpose of MIMIC is to provide a tool for assessing MMS hardware configurations during the mission planning stage or during MMS benchmarking and therefore a standard scenario (the theoretically optimal position of each pulse) is employed. In the following sections the modules for calculating point distribution are detailed.

### 2.1. Profile Angle Module

Changes in profile angle (θ_PrA_) were first explored and quantified in [33]. A method to calculate θ_PrA_ was then detailed and validated for dual-axis scanner rotations and dual-axis target rotations in [34]. θ_PrA_ was calculated through a combination of rotation matrices, 3D surface normals and 2D geometry. For example, in:

(1) $$\text{Rotation Matrix}\left({\text{S}}_{\text{rotated}}\right)={\text{R}}_{\text{x}}\left(\gamma_{\text{scan}}\right){\text{R}}_{\text{y}}\left(\beta_{\text{scan}}\right){\text{R}}_{\text{z}}\left(\alpha_{\text{scan}}\right)$$

**Figure 3.**
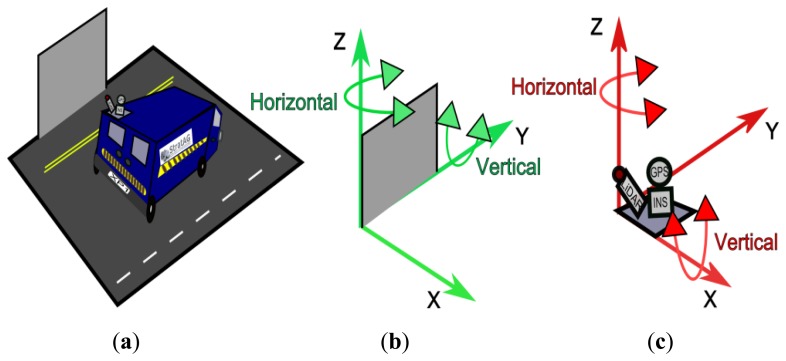
Horizontal and vertical axes of rotation for both target and scanner (**a**) MMS and target (**b**) target axes of rotation (**c**) scanner axes of rotation.

γ, β and α are the vertical, axial and horizontal rotation angles of the scanner. Figure 3 illustrates and Table 1 lists the terminology for the different rotations applied in this paper for both scanner and target. In [34] we showed that this method was susceptible to errors in MMS calibration and also to vehicle dynamics such as pitch, roll and yaw but that accuracy was high and suitable for MMS assessment.

### 2.2. Calculating Profile Spacing

**Figure 4.**
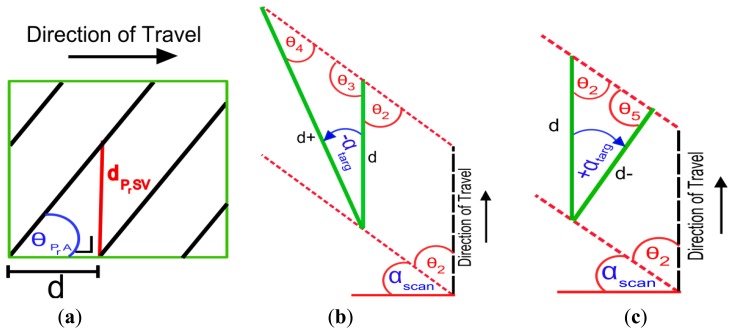
Calculating profile spacing (**a**) profile spacing on a parallel vertical surface (**b**) aerial view of surface rotated away from the MMS-profile spacing increases (**c**) target is rotated towards the MMS-profile spacing decreases.

Although a basic method for calculating profile spacing for dual axis scanner rotations on the road surface was detailed and validated in [35], a more advanced method is required for parallel vertical surfaces or those that have been inclined or rotated (these are referred to as ‘angled’ in this paper). Figure 4a shows a vertical surface with scan profiles striking it. Depending on whether a target is angled towards or away from the MMS, the distance between these scan profiles will decrease or increase. The distance, d is the distance travelled by the vehicle in one mirror rotation and can also be referred to as the horizontal profile spacing, d_PrSH_. For a parallel, vertical target d and d_PrSH_ are identical, but for angled targets they differ. For these formulae, d_PrSH_ is referred to as d+ and d− to illustrate the difference between the direction of rotation of the target. The vertical profile spacing, d_PrSV_ is the vertical distance between scan profiles and is also illustrated in Figure 4a. Calculating profile spacing on an angled surface rotated away from the MMS, d+ in Figure 4b, requires:

(2) $$d+=\frac{d*\sin\theta_{3}}{\sin\theta_{4}}$$

and the profile spacing on an angled surface rotated towards the MMS, d- in Figure 4c, requires:

(3) $$d-=\frac{d*\sin\theta_{2}}{\sin\theta_{5}}$$

To calculate d_PrSV_, the profile angle, θ_PrA_ and the horizontal profile spacing, d_PrSH_ are required. d_PrSV_ can be calculated using:

(4) $$d_{\text{PrSV}}=\frac{d_{\text{PrSH}}*\sin\theta_{\text{PrA}}}{\cos\left(\theta_{\text{PrA}}\right)}$$

### 2.3. Calculating Point Spacing

A method for calculating the point spacing, d_PS_, for the road surface was detailed in [33]. These tests identified that the uncertainty in the road gradient introduced errors into a point spacing calculation that was carried out using planar targets. A novel point spacing calculation method for angled targets is applied in this paper. The first step in computing d_PS_ is calculating the angular step width of the scanner, θ_A_. To find θ_A_, the number of points per mirror rotation, P_p_M, must be identified. Following this, θ_A_ can be found using the FOV of the scanner and the P_p_M:

(5) $$\theta_{\text{A}}=\frac{\text{FOV}}{\text{PpM}}$$

**Figure 5.**
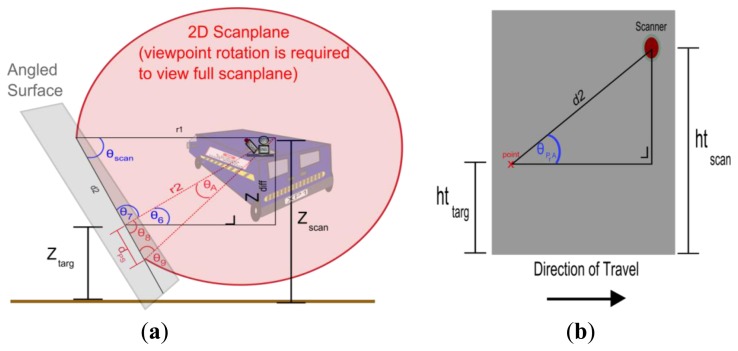
Calculating point spacing (**a**) MIMIC requires an adjusted height of scanner and height of target (**b**) adjusted for vertical rotation of target.

MIMIC calculates d_PS_ through the application of 2D geometric formulae to calculate the distance between subsequent laser pulses on the angled surface, however a horizontal and vertical rotation of the scanner alters the orientation of the scan plane in relation to the target and the scanner. This alters the viewing geometry for the 2D plane and therefore the height of the scanner and the target must be adjusted accordingly in the calculation. Z_scan_ and Z_targ_ represent these adjusted heights in Figure 5a. The amended heights can be calculated using the original target and scanner heights (h_targ_, h_scan_)and the vertical scanner rotation, β_targ_, with:

(6) $$Z_{\text{targ}}=\frac{h_{\text{targ}}}{\cos\left(\gamma_{\text{scan}}\right)}$$

and:

(7) $$Z_{\text{scan}}=\frac{h_{\text{scan}}}{\cos\left(\gamma_{\text{scan}}\right)}$$

The height difference between the scanner and the target, (Z_diff_), is required for these calculations on the 2D plane. The horizontal range to the target, H_r_, must be specified by the user. For this part of the calculation, the target is assumed to be a hypothetical point at the same height as the scanner. Therefore the range from the scanner to the target is r1 in Figure 5a. r1 is dependent on the scanner horizontal rotation, α_scan_ and H_r_. r1 can be calculated with:

(8) $$r1=\frac{H_{r}}{\cos\left(\alpha_{\text{scan}}\right)}$$

Additional variables are required for calculating d_PS_. The next value that is required is the scan angle, θ_scan_. This is the angle formed between r1 and the scan profile on the angled surface, d2. This is calculated in a similar manner to the profile angle as detailed in [34].

Unlike existing methods, MIMIC can incorporate different target heights. Once MIMIC has calculated θ_A_, r1, θ_scan_ and Z_diff_, it can calculate r2. Unlike the horizontal range, H_r_, or the hypothetical range at r1, r2 is the actual range to the target along the scan plane. d2 is required to calculate r2 and is the portion of the scan plane that has intersected with the angled plane between the hypothetical point at r1 and the actual point at h_targ_. Calculating d2 does not require the same rotated perspective that the previous calculation did, but rather one that was adjusted for the vertical rotation of the target only. Therefore ht_scan_ and ht_targ_ are used. Figure 5b illustrates these variables. Once d2 has been calculated the actual range to the target (r2) can be calculated with:

(9) $$r2=\sqrt{d2^{2}+r1^{2}-2\left(\left(d2*r1\right)\left(\cos(\theta_{\text{scan}}\right)\right)}$$

Once r2 has been calculated, the point spacing, d_PS_, can then be found using:

(10) $$d_{\text{PS}}=\frac{\left(r1\right)\left(\sin\theta_{\text{A}}\right)}{\left(\sin\theta_{9}\right)}$$

## 3. Experimental Datasets

Two scenarios involving three datasets were analysed to verify the calculations. A constructed 3D model was used as the control dataset as all external and hardware errors could be eliminated, and point clouds from the MMS designed at the NCG, the XP1, and from a commercial MMS, the Optech Lynx M1 [23], were used to experimentally validate MIMIC's calculations with real-world data.

### 3.1. Dataset 1-Constructed CAD Models

**Figure 6.**
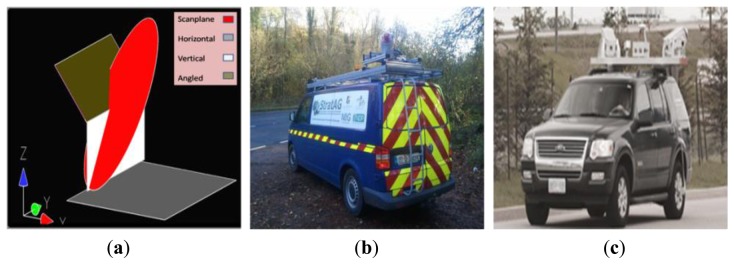
Validation (**a**) initial tests with constructed CAD models (**b**) survey data captured by the XP1 (**c**) survey data captured by the Optech Lynx M1.

For our initial tests, we created a number of planes representing different surfaces and different scanner rotations in Bentley Microstation v8i. Here we measured the interaction between lines, planes and discs. Planes represented targets, discs represented the scan plane and lines represented individual laser pulses. Hardware or configuration issues were therefore absent from these tests. Additionally, external forces such as pitch, roll or yaw of the MMS could not influence the point and profile measurements. Dataset 1 consisted of 14 targets, including horizontal and vertical surface rotations for a dual axis scanner rotation (Figure 6a). We then manually measured the profile spacing and point spacing on each surface for each dual axis scanner rotation in the CAD environment.

### 3.2. Point Cloud Data

Two MMSs were used to validate MIMIC's calculations. The first was the research platform designed by the team at the NCG and the second was using data supplied by Optech Inc. (ON, Canada) from their commercial system, the Optech Lynx M1.

#### 3.2.1. Dataset 2-XP1 MMS

The multi-disciplinary research group StratAG, established to research advanced geotechnologies at NUI Maynooth have designed and developed a multi-purpose land based Mobile Mapping System, the XP1. The primary components of the XP1 (Figure 6b) are an IXSEA LANDINS (Paris, France), a Riegl VQ-250 300 kHz (Horn, Austria) laser scanner and an imaging system consisting of six progressive-scan cameras. Imaging sensors include a thermal camera and a multi-spectral camera capable of sensing across blue, green, red and two infra-red bandwidths. Unlike most commercial systems, the XP1 is a single scanner system.

#### 3.2.2. Dataset 3-Optech Lynx

The commercial system was the Optech Lynx M1 and it provided the opportunity for further validation with different scan hardware and a different system configuration. Unlike the XP1, the Optech Lynx M1 (Figure 6c), is a dual scanner MMS. Each 2D scanner is capable of a 500 kHz PRR and a 200 Hz M_f_. Data from the Optech Lynx data facilitated system verification in a number of ways. Firstly, errors in calibration of the scanners on each MMS could lead to problems measuring target orientations. As it was not possible to verify the orientation of the surfaces using traditional survey methods, the use of two MMS improved the robustness of our tests. Secondly, the dual scanner Optech Lynx was used to verify that MIMIC can cater for dual scanner MMSs as the variations in scanner configuration between the Optech Lynx and the XP1 further verified MIMIC's profile calculations for different system configurations. Finally, the Optech M1 scanner is capable of operating at a higher M_f_ than the Riegl VQ-250 onboard the XP1, providing further test data.

#### 3.2.3. Target Selection and Validation Procedure

For both MMS datasets a number of suitable areas for tests were selected and a series of sample measurements were recorded at each location. Using software developed by researchers at the NCG [36–38], areas consisting of suitable man-made vertical structures (e.g., walls, buildings, roofs, road-side infrastructure) were quickly identified and XP1 survey data was extracted from large files of both rural and urban environments in Ireland. Optech Inc. supplied researchers at the NCG with survey data captured in the vicinity of their offices in Ontario. A set of 16 surfaces were then manually selected from the XP1 and Optech Lynx datasets because by ensuring variation in orientation, range and elevation of the target our calculations could be robustly validated. Each surface had a different elevation in relation to the MMS (±2 m) and was located at between 5 m and 16 m horizontal range to the scanner. Point density varied per target from 300 points to 1,500 points per m^2^. The orientation of each surface varied in horizontal from −60° to +60° and in vertical from 0° to 45°. The range to and orientation of each surface was measured in the point cloud using measuring tools in Bentley Microstation v8i and TerraScan [39]. The inertial measurement units (IMUs) onboard each of the MMSs provided high accuracy velocity and orientation information making it possible to choose scan profiles captured at constant vehicle speeds for profile spacing tests and when vehicle dynamics (*i.e.*, pitch, roll) were minimal. Profile spacing and point spacing were manually measured from the point cloud. The potential for error arose when manual approximation of the scan profile was inhibited by non-uniform point distribution along the scan profile. To assess the impact of this error, five profile spacing measurements were recorded from a single survey for two scan profiles, Profile 1 and Profile 2. Profile 1 displayed non-uniform point distribution while Profile 2 displayed a uniform point distribution. The standard deviation of these measurements (σ) was then calculated. As expected, the profile exhibiting the highest σ (implying a degree of interpretation when delineating a profile) also exhibited the higher errors, 0.003 m as opposed to 0.001 m as summarised in Table 2.

## 4. Results and Discussion

This section details the validation methodology and the results of the profile and point spacing tests. The tests using the constructed models are presented separately to the point cloud tests.

### 4.1. Profile Spacing

To ensure robust validation, MIMIC's profile spacing calculations were first theoretically validated using the constructed CAD models and then using point cloud data from the XP1 and the Optech MMS.

#### 4.1.1. Constructed Models

Vertical surfaces rotated horizontally and vertically around the Y and Z axes respectively were used to validate MIMIC's profile spacing calculations for angled planes. In these initial tests the surface was rotated both clockwise and anti-clockwise around the Z axis. This represented a horizontal rotation of the target in either direction. The same process was applied for rotations around the Y axis which represented inclinations of the target. Combinations of horizontal and vertical rotations were also applied. For these tests a constant vehicle velocity of 10 m/s was applied and a horizontal/vertical dual-axis scanner rotation referred to as 45°/45° was applied. The initial profile spacing measurements return a zero error (Table 3), theoretically validating MIMIC's calculations of profile spacing for angled surfaces with a controlled dataset.

#### 4.1.2. Point Cloud Data

**Figure 7.**
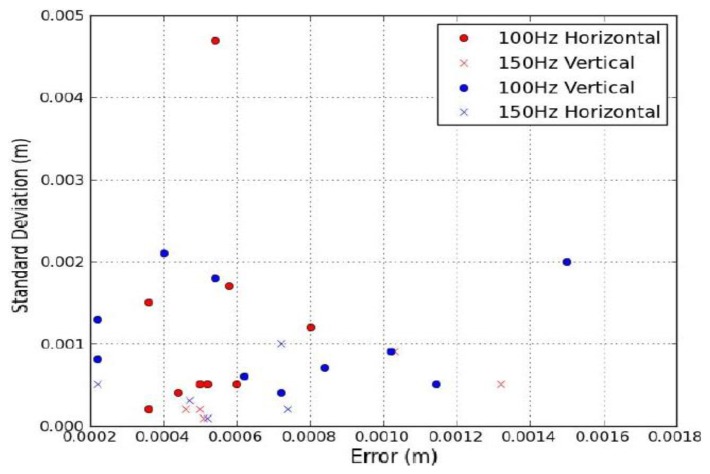
MIMIC's profile spacing calculations: Calculating average error over five measurements plotted against measurement reliability (*σ*) for horizontal and vertical profile spacing at two mirror frequencies −100 Hz and 150 Hz.

Angled structures were selected from the XP1 and Optech Lynx datasets. This enabled validation of MIMIC using point cloud data captured at different vehicle velocities. The Optech Lynx dataset enabled verification of a second scanner configuration and a higher M_f_ than that from the XP1 alone. Data was chosen at two M_f_, 100 Hz and 150 Hz and vehicle velocity varied for each target. Targets 1–6 were surfaces exhibiting pronounced vertical rotations whereas Targets 7–15 focussed on horizontal surface rotations. Five measurements were recorded for both d_PrSH_ and d_PrSV_ for each set of scan profiles. σ was then calculated to provide an indication of the quality of each measurement. The errors from the point cloud tests were higher than the control dataset but were still low, less than 1 mm in all but four of the cases. Figure 7 illustrates the errors and plots them against σ for both horizontal and vertical profile spacing. This helps visualise the quality of the measurements used to validate the calculated values. It can be seen that the majority of MIMIC's calculations have an error of less than 1 mm and a σ of less than 2 mm. The low σ implies that the measurements were reliable, and the low error validates experimentally MIMIC's calculations for profile spacing on angled surfaces at different scanner configurations and vehicle velocities.

### 4.2. Point Spacing

MIMIC's point spacing calculations were also validated using the constructed CAD model as a control dataset and then using the XP1 and Optech point cloud data to assess their performance with real world data.

#### 4.2.1. Constructed Models

Horizontal and vertical target rotations were introduced to verify MIMIC's point spacing calculations for an angled surface. The range from the scanner to the target was fixed but the target orientation was varied. For the initial CAD tests a vertical surface was placed in a selection of orientations and a series of 3D lines representing individual laser pulses were created. The hypothetical scanner was fixed at an elevation of 3.1 m, orientated at 45°/45° and placed at a range of 4 m from the target, although the range varied slightly depending on the rotation of the target. A θ_A_ of 0.12° was applied. The target was rotated in 15° steps horizontally, vertically and a dual axis rotation was also applied. Anti-clockwise and clockwise rotations were introduced. The 3D lines were intersected with the angled surface and the distance between the two points of intersection was measured. The results are displayed in Table 4. The control tests theoretically validate MIMIC's method for calculating point spacing on angled surfaces, returning a zero error.

#### 4.2.2. Point Cloud Data

**Figure 8.**
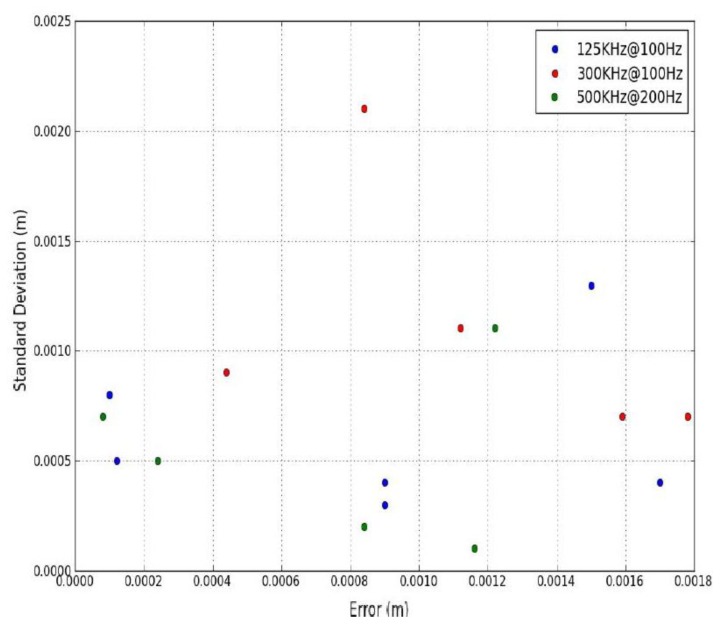
MIMIC's point spacing calculations: Calculating average error over five point spacing measurements plotted against measurement reliablity (*σ)* for three PRRs and two M_f_s.

The distance between subsequent points on the angled surface was measured manually for real-world point cloud data, so this once again introduced a potential error. Point spacing was measured five times on 16 targets and σ calculated to provide an estimate of the quality of the 80 measurements. Targets 1–6 were surveyed using the XP1's Riegl VQ-250 operating at a 300 kHz PRR with a M_f_ of 100 Hz and Targets 7–11 by the Optech Lynx operating at a 125 kHz PRR and with a M_f_ of 100 Hz. Targets 12–16 were surveyed using the same scanner operating at a 500 kHz PRR and a M_f_ of 200 Hz. By altering the target rotation for each test, the range to the target, the height difference between the scanner and target, the PRR and the M_f_, MIMIC's capabilities of predicting point spacing were tested robustly. Figure 8 illustrates the error plotted against σ. The highest σ was approximately 2 mm however, the majority of the measurements displayed less than 1 mm. The largest error was less than 2 mm at a range of approximately 8 m. These results validate MIMIC's calculations using real-world MMS datasets.

### 4.3. Discussion

Table 5 summarises the errors for each of the tests. The largest error is 2 mm, whereas the maximum mean error is 1 mm. This is satisfactory, but for large targets with a low profile spacing an error of 1 mm may impact on the point density calculation due to the high number of profiles. Table 6 has been collated to assist in understanding what may have contributed to MIMIC's calculation errors. Each of the input values was incremented individually until the predicted and measured values match. By examining this table, it is clear that even small discrepancies in specifying input values, measuring target orientation or system settings could account for those errors. Errors could not be quantified for the point spacing tests because an error of 2 mm at 5 m range is more significant than an error of 2 mm at 20 m range to the target.

The versatility and accuracy of the system highlights some of the strengths of the method presented in this paper over existing methods [12,20,22,26–32]. MIMIC's geometric formulae incorporate scanner and target orientation, target and scanner height and all scanner settings. Carrying out a MMS survey and manually interrogating the data is a time consuming process. Additionally, with the manual approach, there is no complete understanding of what factors contribute to point and profile information. Measurements are required for every relevant MMS configuration and target type. To standardise this process, the tests must be performed on the same test-route. Without a powerful workstation, simulators can be slow, although work is ongoing to improve the speed of these systems [40]. Once the simulation is finished, measuring point distribution is a manual process and must be repeated for every area of interest. Simulators are optimal when modelling point distribution for large areas whereas the focus of this paper is on smaller objects, such as individual buildings or roadside infrastructure.

## 5. Conclusions/Outlook

This paper presented the geometric formulae required for calculating the profile spacing and the point spacing on angled targets. The profile spacing and point spacing calculations were validated in a series of tests using constructed CAD models as a control dataset and point cloud data from two MMSs to assess MIMIC's performance with real world data. The first output value from MIMIC was the profile spacing. MIMIC exhibited no error in the CAD tests, thus mathematically validating the profile spacing calculations. The results from the point cloud tests were also promising, with all errors below 2 mm. The highest mean error for the profile spacing tests was 0.001 m. An error of 1 mm is acceptable. The second output value from MIMIC was the point spacing. The control tests using the constructed 3D models once again returned no error, validating MIMIC's point spacing calculations mathematically. The highest mean error for the point spacing tests was 0.001 m, which was encountered in the point cloud tests. Again, an error of 1 mm is acceptable. The main contribution of this paper is a method for calculating point and profile information for different MMS configurations on angled surfaces. It provides a valuable tool for assessing the performance of MMSs, designing survey specifications or tailoring automated algorithms to likely real world scenarios.

## Figures and Tables

**Table 1.** Terminology for horizontal and vertical scanner and target rotations.

**Rotation Axis**	**Target**	**Scanner**
Horizontal	α_targ_	α_scan_
Vertical	β_targ_	γ_scan_

**Table 2.** Profile spacing tests- assessing the reliability of manual profile measurements as a validation tool for MIMIC-the importance of accurately delineating profiles in the point cloud is demonstrated by the positive correlation between σ and error.

**Result**	**Profile 1 (m)**	**Profile 2 (m)**
Measured (Average)	0.109	0.096
MIMIC	0.112	0.095
*σ*	0.008	0.001
Error	0.003	0.001

**Table 3.** Profile spacing-The profile spacing calculated by MIMIC validated using manual measurements on a constructed CAD model for a selection of horizontally rotated (*α_targ_*) and vertically rotated (*β_targ_*) surfaces.

***No***.	***α_targ_***	***β_targ_***	**MIMIC (m)**	**CAD (m)**	**Error (m)**
1	15°	0°	0.082	0.082	0.000
2	0°	15°	0.100	0.100	0.000
3	15°	15°	0.082	0.082	0.000
4	−15°	0°	0.141	0.141	0.000
5	0°	−15°	0.100	0.100	0.000

**Table 4.** Point spacing-The point spacing calculated by MIMIC validated using manual measurements from a constructed CAD model for a selection of horizontally rotated (*α_targ_*) and vertically rotated (*β_targ_*) surfaces. The simulated scanner height was 3.1 m, and a θ_A_ of 0.12° was applied.

***No***.	***α_targ_***	***β_targ_***	**MIMIC (m)**	**CAD (m)**	**Error (m)**
1	15°	0°	0.013	0.013	0.000
2	0°	15°	0.020	0.020	0.000
3	15°	15°	0.016	0.016	0.000
4	30°	0°	0.010	0.010	0.000
5	0°	30°	0.028	0.028	0.000
6	30°	30°	0.021	0.021	0.000
7	−30°	0°	0.034	0.034	0.000
8	0°	−30°	0.006	0.006	0.000
9	−30°	−30°	0.006	0.006	0.000

**Table 5.** Error summary of point and profile tests using constructed CAD models and point cloud data from two real world MMSs.

**Test**	**Output**	**Min (m)**	**Mean (m)**	**Max (m)**
Constructed	*d*_PrSH_	0.000	0.000	0.000
Constructed	*d*_PrSV_	0.000	0.000	0.000
Constructed	*d*_PS_	0.000	0.000	0.000
Real World	*d*_PrSH_	0.000	0.001	0.002
Real World	*d*_PrSV_	0.000	0.001	0.002
Real World	*d*_PS_	0.000	0.001	0.002

**Table 6.** Assessment of errors for the horizontal and vertical profile spacing tests by quantifying the uncertainties in any of the input parameters that might account for errors in the real world tests.

**Surface**	**Err (m)**	***α_scan_***	***γ_scan_***	***α_targ_***	***β_targ_***	***M_f_***	**Velocity (m/s)**
*d*_Pr SH_	0.002	1.50°	n/a	1.93°	n/a	4.00 Hz	0.200
*d*_Pr SV_	0.002	1.00°	0.5°	n/a	1°	1.50 Hz	0.130
